# Dexamethasone Suppression Testing in Patients with Adrenal Incidentalomas with/Without Mild Autonomous Cortisol Secretion: Spectrum of Cortisol Cutoffs and Additional Assays (An Updated Analysis)

**DOI:** 10.3390/biomedicines13092169

**Published:** 2025-09-05

**Authors:** Alexandra-Ioana Trandafir, Mara Carsote

**Affiliations:** 1 PhD Doctoral School of “Carol Davila” University of Medicine and Pharmacy, 020021 Bucharest, Romania; 2 Department of Endocrinology, “Carol Davila” University of Medicine and Pharmacy, 020021 Bucharest, Romania; 3 Department of Clinical Endocrinology V, “C.I. Parhon” National Institute of Endocrinology, 011863 Bucharest, Romania

**Keywords:** endocrine neoplasia, hormone, endocrine testing, cortisol, ACTH, hypertension, diabetes, endocrine tumor, adrenal, cutoff

## Abstract

**Background/Objective**: The overnight 1-mg dexamethasone suppression test (DST) represents the conventional/standard tool for endogenous hypercortisolemia screening, typically in relationship with adrenal and pituitary masses. Nevertheless, an associated spectrum of challenges and pitfalls is found in daily practice. This analysis aimed to evaluate: (I.) the diagnosis relevance of 1-mg DST in patients with adrenal incidentalomas (AIs) with/without mild autonomous cortisol secretion (MACS) exploring different cutoffs of the second-day plasma cortisol after dexamethasone administration (cs-DST) with respect to cardio-metabolic outcomes; (II.) the potential utility of adding other biomarkers to DST [plasma morning adrenocorticotropic hormone (ACTH), 24-h urinary free cortisol (UFC), late-night salivary cortisol (LNSC), dehydroepiandrosterone sulfate (DHEAS)]; and (III.) DST variability in time. **Methods**: This narrative analysis was based on searching full-text, English articles in PubMed (between January 2023 and April 2025) via using different term combinations: “dexamethasone suppression test” (*n* = 239), “diagnosis test for autonomous cortisol secretion” (*n* = 22), “diagnosis test for mild autonomous cortisol secretion” (*n* = 13) and “diagnosis test for Cushing Syndrome” (*n* = 61). We manually checked the title and abstract and finally included only the studies that provided hormonal testing results in adults with non-functional adenomas (NFAs) ± MACS. We excluded: reviews, meta-analyses, editorials, conference abstracts, case reports, and case series; non-human research; studies that did not provide clear criteria for distinguishing between Cushing syndrome and MACS; primary aldosteronism. **Results**: The sample-focused analysis (*n* = 13 studies) involved various designs: cross-sectional (*n* = 4), prospective (*n* = 1), retrospective (*n* = 7), and cohort (*n* = 1); a total of 4203 patients (female-to-male ratio = 1.45), mean age of 59.92 years. I. Cs-DST cutoffs varied among the studies (*n* = 6), specifically, 0.87, 0.9, 1.2, and 1.4 µg/dL in relationship with the cardio-metabolic outcomes. After adjusting for age (*n* = 1), only the prevalence of cardiovascular disease remained significantly higher in >0.9 µg/dL vs. ≤0.9 group (OR = 2.23). Multivariate analysis (*n* = 1) found cs-DST between 1.2 and 1.79 µg/dL was independently associated with hypertension (OR = 1.55, 95%CI: 1.08–2.23, *p* = 0.018), diabetes (OR = 1.60, 95%CI: 1.01–2.57, *p* = 0.045), and their combination (OR = 1.96, 95%CI:1.12–3.41, *p* = 0.018) after adjusting for age, gender, obesity, and dyslipidemia. A higher cs-DST was associated with a lower estimated glomerular filtration rate (eGFR), independently of traditional cardiovascular risk factors. Post-adrenalectomy eGFR improvement was more pronounced in younger individuals, those with lower eGFR before surgery, and with a longer post-operative follow-up. Cs-DST (*n* = 1) was strongly associated with AIs size and weakly associated with age, body mass index and eGFR. Cortisol level increased by 9% (95% CI: 6–11%) for each 10 mL/min/1.73 m^2^ decrease in eGFR. A lower cs-DST was associated with a faster post-adrenalectomy function recovery; the co-diagnosis of diabetes reduced the likelihood of this recovery (OR = 24.55, *p* = 0.036). II. Additional biomarkers assays (*n* = 5) showed effectiveness only for lower DHEAS to pinpoint MACS amid AIs (*n* = 2, cutoffs of <49.31 µg/dL, respectively, <75 µg/dL), and lower ACTH (*n* = 1, <12.6 pmol/L). III. Longitudinal analysis of DST’s results (*n* = 3): 22% of NFAS switch to MACS after a median of 35.7 months (*n* = 1), respectively, 29% (*n* = 1) after 48.6 ± 12.5 months, 11.8% (*n* = 1) after 40.4 ± 51.17 months. A multifactorial model of prediction showed the lowest risk of switch (2.4%) in individuals < 50 years with unilateral tumor and cs-DST < 0.45 µg/dL. In the subgroup of subjects without cardio-metabolic comorbidities at presentation, 25.6% developed ≥1 comorbidities during surveillance. **Conclusions**: The importance of exploring the domain of AIs/NFAs/MACS relates to an increasing detection in aging population, hence, the importance of their optimum hormonal characterization and identifying/forestalling cardio-metabolic consequences. The spectrum of additional biomarkers in MACS (other than DST) remains heterogeneous and still controversial, noting the importance of their cost-effectiveness, and availability in daily practice. Cs-DST serves as an independent predictor of cardio-metabolic outcomes, kidney dysfunction, while adrenalectomy may correct them in both MACS and NFAs, especially in younger population. Moreover, it serves as a predictor of switching the NFA into MACS category during surveillance. Changing the hormonal behavior over time implies awareness, since it increases the overall disease burden.

## 1. Introduction

Dexamethasone, a potent synthetic glucocorticoid, is routinely used to assess the hypothalamic-pituitary-adrenal (HPA) axis in daily endocrine practice [1]. The 1-mg (overnight) dexamethasone suppression test (DST) is a conventional tool that evaluates endogenous hypercortisolemia, typically in adrenal and pituitary masses; being regarded as a standard assessment, despite controversies are still found in everyday testing [1,2,3]. Patients with normal HPA function have post-DST (second day) morning cortisol levels of 1.8 μg/dL or lower [2,4], which reflects a normally suppressed cortisol, with a 98.6% sensitivity and 90.6% specificity [5]. A serum cortisol level above 1.8 µg/dL implies a positive test, indicating either (endogenous) Cushing’s syndrome or mild autonomous cortisol secretion (MACS) [6].

Over time, several diagnosis criteria for MACS have been proposed, being centered on the lack of serum cortisol suppression amid DST with various cutoffs (currently, being agreed for a value higher than 1.8 and lower than 5 µg/dL, in the absence of the clinical phenotype highly suggestive for Cushing’s syndrome). 1-mg DST is the mostly used dosage for the primary screening, alternatively, 2 days of 2 mg dexamethasone testing may be applied in selected cases (0.5 mg of dexamethasone 6-hourly for 48 h, with blood cortisol assays at baseline and after 48 h), being named low-dose dexamethasone suppression test (LDDST) [7,8,9].

In addition, the diagnosis of MACS might be suggested by a decreased or low-normal basal morning plasma adrenocorticotropic hormone (ACTH), an elevated midnight serum cortisol or late-night salivary cortisol (LNSC), and/or an increased urinary free cortisol (UFC), which otherwise are not currently regarded as standard diagnosis criteria, as opposite to DST [9,10]. Moreover, a low dehydroepiandrosterone sulfatelevel (DHEAS) may help MACS recognition [11].

Advancements in imaging tools, increased utilization of different imaging procedures, and prolonged life span in certain populations had generally contributed to a rise in the detection of incidentalomas, including at the level of adrenal glands (AIs) [12,13]. AIs prevalence increases with age, ranging from 3% by the age of 50 years to 10% in adults over 70 years, but the true epidemiologic impact remains an open matter [14,15,16].

MACS-positive profile represents the most common hormonal abnormality in AIs, affecting from 1% to 29–35% of all these tumors (the ranges depend on the diagnosis criteria and the characteristics of the study population) [17,18,19,20]. Patients with MACS are at higher risk [versus (vs.) general population] for cardio-metabolic disorders, including hypertension, obesity, glucose intolerance or type 2 diabetes, dyslipidemia, and secondary renal complications [21,22,23], respectively, osteopenia/osteoporosis, and osteoporotic fractures with impaired fracture healing [24,25,26,27]. More recently, emerging data suggested that even individuals with AIs who were classified as non-functioning adrenal adenomas (NFA) may still carry a higher risk of diabetes, hypertension, and associated cardiovascular events compared to those without any adrenal tumor. Thus, it suggests some limits of the current assays/criteria in order to address the true hormonal burden of this adrenal neoplasia [28,29,30]. Moreover, MACS has been linked to a higher mortality, mostly because it increases the risk of cardiovascular events, hence, the importance of an adequate hormonal characterization of these tumors [31,32].

Furthermore, an adequate lab and imaging diagnosis should be followed by an adequate management. However, the most effective treatment for MACS-positive neoplasia embraces a large spectrum, varying from long term conservative approach (while managing MACS-related comorbidities, e.g., diabetes, hypertension, osteoporosis, etc.); “wait-and-see” approach (meaning to indicate surgery in case of a novel complication or worsening of the baseline clinical features e.g., new fractures, lack of response to usual anti-hypertensive or anti-diabetic drugs); adrenalectomy decision solely based on the tumor size (e.g., larger than 4 cm) or adrenal removal in each MACS-positive patient [31,32,33].

### Objective

This analysis aimed to evaluate the diagnosis relevance of 1-mg DST in patients with NFAs/MACS exploring different cutoffs of the second-day morning plasma cortisol after dexamethasone administration with respect to the cardio-metabolic outcomes, the importance of adding supplementary biomarkers to DST such as plasma morning ACTH, 24-h UFC, LNSC, DHEAS, as well as DST variability over time (e.g., switch between the three categories: NFA, MACS, or overt Cushing syndrome).

## 2. Methods

This narrative review was conducted based on a targeted literature search of full-text, English-language articles available in PubMed via using the following terms: “dexamethasone suppression test” (*n* = 239), “diagnosis test for autonomous cortisol secretion” (*n* = 22), “diagnosis test for mild autonomous cortisol secretion” (*n* = 14) and “diagnosis test for Cushing Syndrome” (*n* = 61). We included only original studies published between January 2023 and April 2025. We manually checked the title and abstract and finally included only the articles that provided hormonal testing results in NFAs/MACS. We excluded: reviews, meta-analyses, editorials, conference abstracts, case reports, and case series; non-English papers; pediatric cohorts; non-human research; studies that did not provide clear criteria for distinguishing between Cushing syndrome and MACS; study population diagnosed with primary aldosteronism.

**Figure 1:**
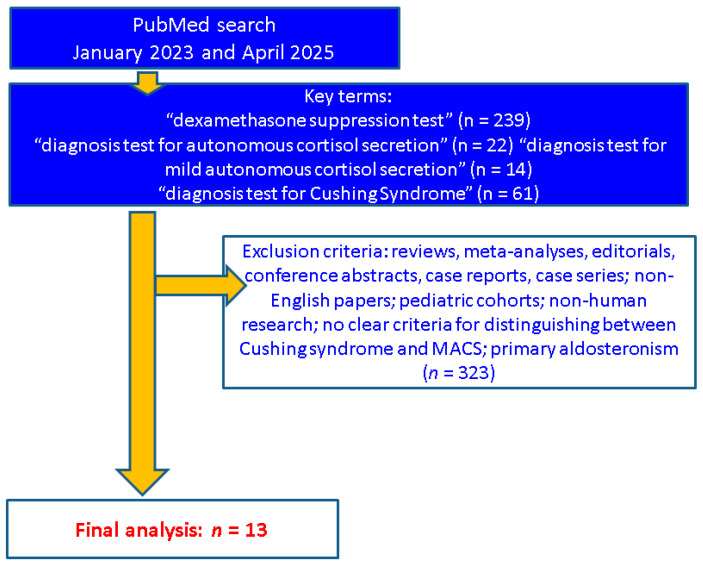
Flowchart diagram of search (*n* = number of studies).

The final sample-focused analysis (*n* = 13 studies) involved various designs: cross-sectional (*n* = 4), prospective (*n* = 1), retrospective (*n* = 7), and cohort (*n* = 1); a total of 4203 patients (female-to-male ratio of 1.45), mean age of 59.92 years (Figure 1).

## 3. Sample-Focused Analysis

### 3.1. Post-DST Cortisol Cutoffs in Relationship with the Spectrum of Comorbidities

Post-DST cortisol cutoffs varied among the studies, being reflected in different risk ratios for cardio-metabolic and renal complications. Overall, we identified six studies to address this specific issue, noting the following cutoffs (in addition to the standard value of 1.8 µg/dL) 0.87, 0.9, 1.2 and 1.4 µg/dL [34,35,36,37,38,39] (Table 1).

In patients diagnosed with MACS-positive tumors, higher post-DST cortisol levels have been correlated with an increased prevalence of cardiovascular risk factors, and drug-resistant hypertension [40,41], and even an increased mortality (in some cohorts) [42]. A blood cortisol value above 0.9 μg/dL was associated with an elevated risk of developing at least one cortisol excess-related comorbidity (e.g., hypertension, diabetes mellitus, or osteoporotic fragility fractures, etc.) [43]. This post-DST threshold of 0.9 µg/dL was investigated, for instance, by Araujo-Castro et al. [38]: a total of 593 NFAIs were included, and 412 patients had post-DST cortisol ≤ 0.9 µg/dL (69.5%), while 181 subjects had post-DST cortisol > 0.9 µg/dL (30.5%). After adjusting for age, only the prevalence of cardiovascular disease remained significantly higher in >0.9 µg/dL vs. ≤0.9 group [adjusted odds ratio of (OR) of 2.23] [38].

Another cutoff was investigated by Favero et al. [37] who concluded that hypertension, diabetes, and their combination were statistically significant associated with a post-DST cortisol cutoff above 1.2 µg/dL that brings the highest accuracy in identifying patients with either hypertension or diabetes [area under the curve (AUC) of 0.604; 95% confidence interval (CI) between 0.560 and 0.649; sensitivity of 60.2%, specificity of 56.0%] or hypertension plus diabetes (AUC of 0.611, 95% CI between 0.545 and 0.675, sensitivity 60.4%, specificity 69.6%). Patients with a cortisol level between 1.2 and 1.79 µg/dL (N = 326) vs. < 1.2 µg/dL (N = 289) showed lower baseline ACTH (15.3 ± 10.1 vs. 17.7 ± 11.9 pg/mL, *p* = 0.008), were older (62.5 ± 10.9 vs. 57.5 ± 12.3 years, *p* < 0.001), and had higher rates of hypertension (52.5% vs. 38.1%, *p* < 0.001), diabetes (23.3% vs. 13.1%, *p* = 0.001), combined hypertension and diabetes (16.9% vs. 8.3%, *p* < 0.002), as well as cardiovascular events (7.3% vs. 3.2%, *p* = 0.028). Multivariate analysis confirmed that cortisol levels between 1.2 and 1.79 µg/dL were independently associated with hypertension (OR = 1.55, 95% CI: 1.08–2.23, *p* = 0.018), diabetes (OR = 1.60, 95% CI: 1.01–2.57, *p* = 0.045), and their combination (OR = 1.96, 95% CI: 1.12–3.41, *p* = 0.018) after adjusting for age, gender, obesity, and dyslipidemia [37].

Güneş et al. [39] explored ROC-based cut-offs by including 123 patients with AIs and 114 age- and sex-matched controls with thyroid nodules with similar age (53.0 ± 10.9 vs. 52.9 ± 7.4 years, *p* = 0.98) and gender distribution (female-to-male ratio of 90/33 vs. 91/23, *p* = 0.28). The prevalence of hypertension was higher in AIs group vs. controls (50.4% vs. 31.6%, *p* = 0.004). ROC analysis showed the optimal post-DST cortisol linked to the hypertension diagnosis was of 0.87 μg/dL: subjects with a level < 0.87 μg/dL had a lower frequency of hypertension vs. ≥ 0.87 μg/dL (42.6% vs. 66.1%, *p* = 0.009), with binary logistic regression analysis identifying age (β = 0.068, OR = 1.07, 95% CI: 1.02–1.12, *p* = 0.004) and post-DST cortisol value (β = 1.18, OR = 3.24 95% CI: 1.02–10.34, *p* = 0.047 as independent predictors for hypertension [39].

Notably, MACS-positive tumors might affect kidney function in various ways since comorbidities such as hypertension, diabetes, and dyslipidemia may lead to glomerular damage, albuminuria, and proteinuria [44]. Chronic cortisol over-production directly alters the renal vascular tone, by increasing the vascular resistance and thus contributing to hypertension development. Furthermore, fluid retention and hypertension arises from increased salt and water reabsorption in renal tubules due to the activation of the mineralocorticoid receptor by excessive cortisol. Chronic kidney disease is connected to the over-activation of the mineralocorticoid receptor, which is present in renal tubular cells, as well as endothelial cells, podocytes, and fibroblasts [45]. Rahimi et al. [36] found that higher post-DST cortisol was associated with lower estimated glomerular filtration rate (eGFR), independently of traditional cardiovascular risk factors. The cohort included 972 individuals (44% of them had MACS, and 56% were classified as NFAs). At diagnosis, patients with MACS showed a statistically significant lower eGFR vs. NFAs (79.6 mL/min/1.73 m^2^ vs. 83.8 mL/min/1.73 m^2^, *p* < 0.001). Multivariable analysis showed that post-DST cortisol was an independent predictor of kidney function. Specifically, each doubling of post-DST cortisol level was associated with a decrease of 1.01 mL/min/1.73 m^2^ in eGFR (*p* = 0.017). Alongside cortisol levels, older age (−7.94 mL/min/1.73 m^2^; *p* = 0.001) and hypertension (−2.72 mL/min/1.73 m^2^; *p* = 0.038) were also independently associated with a decreased eGFR. The group of subjects who underwent adrenalectomy [N = 204, including 155 patients with MACS (76%) and 49 people with NFAs (24%)] showed a gradual eGFR improvement (in both subgroups), starting at 18 months up to 3.5 years after surgery. This correction was more pronounced in younger individuals, people with lower eGFR before adrenalectomy, and those with a longer post-operative follow-up. Overall, these findings suggested that post-DST cortisol might serve as an independent predictor of a kidney dysfunction, while adrenalectomy may correct the renal status in both MACS and NFAs, especially in younger population [36].

Olsen et al. [35] (N = 1129 patients with AIs) found that post-DST cortisol was strongly associated with AIs size and weakly associated with age, body mass index (BMI), and eGFR. Cortisol level increased by 9% (95% CI: 6–11%) for each 10 mL/min/1.73 m^2^ decrease in eGFR. Additionally, the cortisol value showed a nonlinear relationship with BMI (cortisol decreasing in cases with BMI below 30 kg/m^2^, and remaining unchanged at higher BMI values). The study also revealed that post-DST cortisol increased by 11% (95% CI: 7–14%) for each 10-year increase in age and by 23% (95% CI: 16–31%) for each 5 kg/m^2^ decrease in BMI for those subjects with a BMI < 30 kg/m^2^ [35].

Higher pre-operatory values of post-DST cortisol were correlated with lower post-adrenalectomy cortisol in addition to the co-presence of the mentioned comorbidities that potentially improve after tumor removal [44,45,46,47,48,49]. A lower preoperative cortisol level after 1-mg DST was statistically significant associated with a faster HPA axis recovery (*p* < 0.001) in one study. Post-DST cortisol was the only biochemical predictor of 6-week recovery regarding post-adrenalectomy adrenal insufficiency, with a threshold of 4.75 µg/dL yielding 89.5% sensitivity and 72.7% specificity (AUC = 0.87, 95% CI: 66.9–98.7, *p* < 0.001). In contrast, baseline morning ACTH and other clinical variables were not predictive for this recovery. However, the co-diagnosis of diabetes mellitus statistically significant reduced the likelihood of the adrenal failure recovery (OR = 24.55, *p* = 0.036) [34].

On short note, post-DST cortisol remains a valuable tool in predicting the panel of MACS-related comorbidities, particularly, of cardio-metabolic type, and other cutoffs than the traditional value of 1.8 µg/dL are under evaluation for their predictive power, while the vast domain of these hormonal assays remains an open issue which we expect to change in the future, probably as multimodal algorithms of diagnosis and further on as a tailored decision-making (conservative vs. surgical).

### 3.2. DST and Additional Assays

The spectrum of additional tests for the diagnosis of endogenous hypercortisolism (other than DST) is heterogeneous and still controversial, noting the importance of their cost-effectiveness, and their availability in daily practice. We identified five studies that analyzed the usefulness of assessing baseline morning ACTH, UFC, LNSC, and DHEAS) [50,51,52,53,54] (Table 2).

#### 3.2.1. Baseline Morning Blood ACTH

ACTH assays are commonly used to differentiate between adrenal Cushing’s syndrome Cushing’s disease, but previous studies have not established a definitive cutoff. Efthymiadis et al. [51] identified the value of 12.6 pmol/L as the optimal cutoff, which effectively distinguishes between the two etiologies with high sensitivity and specificity (AUC = 1.00). Of note, the authors evaluated the diagnosis performance of several screening tests for MACS (in association with low ACTH): LNSC (cutoff 1.7 nmol/L), LNSE (cutoff 15.2 nmol/L), and DST (1.8 µg/dL). They individually showed comparable performance in identifying endogenous hypercortisolism, with DST demonstrating the best results (AUC = 0.76, sensitivity 100%, specificity 52.2%), followed by LDDST (AUC = 0.83, sensitivity 93.8%, specificity 72.7%), LNSC (AUC = 0.71, sensitivity 77.2%, specificity 64.8%). In contrast, UFC showed only a limited diagnosis value (AUC = 0.62), as well as LNSE (AUC = 0.66) [51].

#### 3.2.2. Urinary Steroid Profile

Recently, analyzing the steroids spectrum in plasma and urine through liquid chromatography-tandem mass spectrometry (steroid metabolomics) has emerged as a valuable method for supporting the diagnosis of MACS, as used in other adrenal and pituitary ailments and glucocorticoids exposure [55,56,57,58,59]. These biomarkers that reflect the glucocorticoid excess could indicate a higher risk of cardio-metabolic diseases in AIs [60,61,62]. Although urinary steroid profile does not help in differentiating between benign and malignant adrenal tumors, it might serve as an early marker of MACS/ACS and for identifying AIs subgroups with increased cardio-metabolic risk [63,64]. Araujo-Castro et al. [53] analyzed 49 patients with AIs (25 subjects diagnosed with MACS and 24 with NFAs), and found than the excretion of various glucocorticoid metabolites, such as β-cortolone, α-cortolone, α-cortol, tetrahydrocortisol (THF), tetrahydro-11-deoxycortisol (THS), and tetrahydrocortisone (THE), was elevated in AIs. Except for β-cortolone, which was more frequently increased in subjects with MACS vs. NFAs (16% vs. 0%, *p* = 0.041), the rate of patients with elevated concentrations of these metabolites was equal in MACS vs. NFA. Among these, THS and THF showed the strongest correlation with post-DST cortisol (*r* = 0.548, *p* < 0.001, and, *r* = 0.441, *p* = 0.002, respectively), while overall glucocorticoid metabolite excretion showed a moderate positive correlation (*r* = 0.401, *p* = 0.004). MACS-related comorbidities were moderately accurately predicted by the post-DST cortisol alone (AUC = 0.767, 95% CI: 0.634–0.882). The combination of post-DST cortisol with urinary cortisone, α-cortolone and THS had the highest diagnosis accuracy for MACS-associated complications (AUC = 0.813 95% CI: 0.680–0.912). Nevertheless, the diagnosis efficacy was greatly enhanced by adding serum DHEAS (AUC = 0.853, 95% CI: 0.712–0.954) [53].

#### 3.2.3. Salivary Cortisone

Salivary cortisone has been found as a better diagnosis test than salivary cortisol in adrenal insufficiency [65,66]. Saliva contains more cortisone than cortisol due to the cortisol conversion to cortisone by 11 beta-hydroxysteroid dehydrogenase 2 [67]. Some studies suggested that salivary cortisone has a stronger and more linear correlation with serum total cortisol and serum free cortisol than salivary cortisol [68]. We identified the study of Issa et al. [54] that evaluated the use of salivary cortisone as a non-invasive alternative to 1-mg DST. A strong correlation between salivary cortisone and serum cortisol after administering 1 mg dexamethasone was confirmed (*r* = 0.95, *p* < 0.001). Overall, the diagnosis performance using four predictive parameters: post-dexamethasone salivary cortisone, baseline serum cortisol, salivary cortisone suppression ratio (pre-/post-dexamethasone), and sex yielded a sensitivity of 88.5%, specificity of 91.2%, and a kappa coefficient of 0.80. A simplified model using only post-dexamethasone salivary cortisone displayed similar results (sensitivity 85.3%, specificity 91.7%, kappa 0.77), suggesting that it may be a reliable standalone marker for identifying individuals with serum cortisol ≤ 1.8 µg/dL after 1-mg DST [54].

#### 3.2.4. DHEAS

DHEAS assay might help in MACS identification, DHEAS being an androgen precursor secreted by the adrenal zona reticularis under the dominant regulation of ACTH, thus a relative DHEAS suppression has been described in this type of adrenal neoplasia when accompanied by low or low-normal baseline ACTH levels [69,70]. However, the sensitivity and specificity of DHEAS assays varied among studies. For instance, we mention a retrospective study of Erdogan et al. [50] in 461 patients with AIs (77 with MACS and 384 with NFAs): MACS associated an increased risk of cardio-metabolic diseases vs. NFAs, and one of the most important independent predictors of MACS was a low DHEAS level (≤49.31 µg/dL), which demonstrated a good diagnosis performance (61% sensitivity and 73% specificity). In addition to reduced DHEAS, a larger tumor and the presence of bilateral adrenal masses were suggestive for a MACS-positive profile. On the other hand, ROC analysis revealed that morning plasma cortisol after DST was not a statistically significant predictor of diabetes, hypertension, hyperlipidemia, or coronary artery disease within the MACS subgroup (*p* > 0.05) [50].

Moreover, Al-Waeli et al. [52] reported a different DHEAS cutoff of 75 µg/dL or less, achieving a higher sensitivity (80%) and specificity (73.3%) for MACS identification (14% of the cohort included MACS-positive tumors). ROC curve analysis supported the diagnosis value of this cutoff (AUC = 0.78, 95% CI: 0.57–0.98, *p* = 0.04), although the positive predictive value was modest (33.3%), with a negative predictive value of 95.7%. A statistically significant correlation between MACS and a DHEAS cutoff level of 75 µg/dL or less and a DHEAS ratio of 1.7 or less (*p* = 0.02 and 0.01, respectively) was confirmed. Yet, DHEAS levels were similar in MACS vs. NFAs (75.9 ± 75.3 µg/dL vs. 243.1 ± 264.4 µg/dL, *p* = 0.1) [52].

### 3.3. DST Results: Variations During Long-Term Surveillance

When evaluating newly identified patients with an adrenal mass, the primary goals are to determine whether the lesion is benign or malignant and to evaluate the functionality/hormonal panel [71,72]. During follow-up, the hormonal spectrum and imaging presentation might change, yet, there is a current gap in accurately predicting the behavior in each AI case (which is not primarily referred to surgery) across life span. For instance, a tumor showing NFA profile and a diameter larger than 4 cm displays a two-fold increase risk of MACS transformation during surveillance, according to some data [73]. A higher post-DST level (but remaining below 1.8 µg/dL) might predict a switch from NFA to MACS category after three to four years since initial diagnosis [74,75]. Approximately 10% of NFAs may develop ACS/MACS profile during follow-up [76]. According to our methods, we identified three studies [38,77,78] to address the longitudinal component of DST (one of them has already been mentioned [38]) (Table 3).

In one study, a subgroup of 73 subjects (representing 22.1% out of 331 patients with NFAs) switched to a MACS-positive profile (defined as post-DST cortisol > 1.8 µg/dL) over a median follow-up of 35.7 months. MACS incidence rates were correlated to post-DST cortisol levels at first evaluation, occurring at 19.2, 32.3, and 68.1 cases per 10,000 person-years for cortisol values of <0.9 µg/dL, 0.9–1.3 µg/dL, and >1.3 µg/dL, respectively. These findings suggested that individuals with a cortisol value below 0.9 µg/dL are at very low risk of switching to MACS and follow-up is probably unnecessary. Subjects with intermediate levels (0.9–1.3 µg/dL) represent a moderate-risk group and may benefit from individualized follow-up strategies, consider repeating DST every two-three years for five years after original diagnosis. Moreover, patients exceeding the level of 1.3 µg/dL require annual re-evaluation for at least five years [78].

The most effective predictive model for MACS incorporated various parameters such as age, post-DST cortisol, and the presence of bilateral adrenal tumors, showing good diagnosis accuracy (AUC = 0.70, 95% CI: 0.65–0.75). The lowest risk of switching into MACS (2.42%) category was observed in patients under 50 years with unilateral adrenal neoplasia and post-DST cortisol below 0.45 µg/dL. Baseline post-DST cortisol was the strongest predictor of progression to MACS-positive status, with a hazard ratio of 3.56 per µg/dL increase (*p* < 0.001). Additionally, these patients had lower blood ACTH and DHEAS compared to those who remained with stable hormone levels/same category (13.4 ± 8.13 vs. 16.9 ± 12.5 mg/dL, *p* = 0.038, and 433.7 ± 289.1 vs. 758.3 ± 856.2 µg/dL, *p* = 0.025, respectively). There were no statistically significant differences in UFC (*p* = 0.404) or LNSC (*p* = 0.379) between these two groups. During follow-up, 4.4% of the subjects developed diabetes, 11.5%–hypertension, 24.3%–dyslipidemia, and 4.6%–obesity. 1.2% of the individuals experienced novel cardiovascular events and 0.4% had cerebrovascular events. In the subgroup of subjects without cardio-metabolic comorbidities at presentation, 25.6% of them developed one or more comorbidities that were connected to the adrenal profile. There was a similar risk of developing cardio-metabolic complications between patients with NFAs who progressed to MACS and those who remained NFAs during surveillance [78].

Another study in 132 patients with AIs (56 men and 76 women; the mean age was of 61.7 ± 10.8 years) assessed clinical abnormalities at baseline and during a mean follow-up of 48.6 ± 12.5 months. Patients underwent evaluation of demographic, anthropometric, biochemical, metabolic, and hormonal data, as well as the 24-h ambulatory blood pressure monitoring. At baseline, subjects with MACS (post-DST cortisol of >1.8 µg/dL) showed a higher diastolic blood pressure, glycaemia, and uric acid levels compared to those with a negative DST. During follow-up, 29% of patients initially classified with NFA developed MACS, although cardiovascular and metabolic changes were less pronounced than in individuals diagnosed with MACS from the start [77]. In another study, 11.8% of the subjects with AIs developed MACS after an average follow-up of 40.4 ± 51.17 months. This probability of evolution was increased in persons with higher post-DST cortisol levels at diagnosis (HR = 6.45 for each µg/dL, *p* = 0.001), an increased risk of switching to MACS being found if cortisol assay exceeded 1.4 µg/dL [38].

To conclude, post-DST cortisol levels, the panel of comorbidities, patient’s life expectancy, and health resources should all be taken into account in a customized follow-up of AIs, particularly in non-surgery candidates, since potentially a switch of category (from NFA to MACS) might be found. Awareness is required to check once again the cardio-metabolic spectrum during longitudinal observations and decide adrenalectomy in selected cases. Most probably, the model de prediction should be multifactorial, while post-DST cortisol level represents, on one hand, the core of the clinical decision at a certain point in time, and, on the other hand, the acknowledgment of further developing cardio-metabolic consequences, noting that other cutoffs than the traditional one of 1.8 µg/dL might help for a multimodal decision. Overall, the cross-sectional cortisol assay of less than 1.8 µg/dL amidst DST in a newly identified individual with AI might imply a completely distinct meaning if the value is very low (e.g., <0.9 µg/dL) or very close to the 1.8 µg/dL cutoff in terms of associated cardiovascular and metabolic ailments in time and the risk of switching to an active hormonal activity (e.g., MACS-positive profile) [38,77,78].

## 4. Discussion

**Figure 2:**
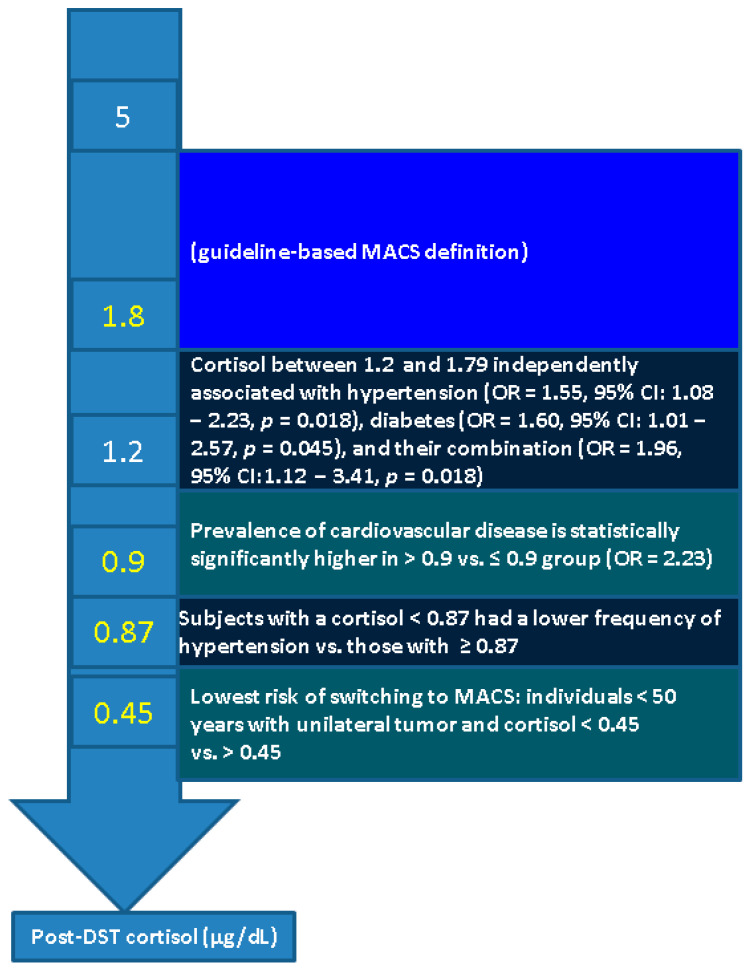
Sneak peek of post-DST cortisol cutoffs and their significance amid the sample-based analysis (Abbreviations: CI = confidence interval, DST = dexamethasone suppression test, MACS = mild autonomous cortisol secretion, OR = odd ratio).

Based on our methods, we provided a narrative analysis starting from the results of thirteen studies that addressed DST from various points of view (applying new cortisol cutoffs, the panel of associated cardio-metabolic outcomes, multifactorial models of behavior prediction, and long-term surveillance). DST seems to associate a heterogeneous spectrum of gaps, while the test represents the current (guideline-based) standard [34,35,36,37,38,39,50,51,52,53,54,77,78] (Figure 2).

### 4.1. The Spectrum of DST: Challenges and Pitfalls

Generally, DST accuracy is influenced by numerous factors causing false positive or negative results [79,80,81]. False positive results might occur in relationship with obesity, anorexia, chronic alcohol consumption, psychiatric illness, advanced age, oral contraception, and use of CYP3A4 inhibitors (which enhance the dexamethasone bioavailability) or in conditions causing a reduced cortisol-binding globulin, such as proteinuria [82,83,84,85,86]. A suboptimal dexamethasone concentration may cause false positive results at rates ranging from 6% to 20% [87,88,89]. For instance, in one study on MACS-positive tumors, 11% of DST’s outcomes were invalid due to achieving only low dexamethasone levels, as found upon an incorrect intake, concurrent use of glucocorticoids or CYP3A4-inhibiting drugs, concomitant gastrointestinal conditions (e.g., vomiting, diarrhea), etc. [90]. In addition, immunoassay-related biases and inter-assay variability might impact the test results [91].

Globally, the aging population is associated with an increased prevalence of AIs that requires DST at diagnosis and during monitoring. Despite physiological changes that occur with age such as altered drugs metabolism, small alterations of HPA axis sensitivity, and cortisol rhythm, DST relies on a standard dose for all adults, which actually might require refining the dose in selected subgroups, for instance, in elderly with obesity, etc. [92,93,94,95]. Currently, urinary metabolomics has been explored as an additional screening method in endogenous hypercortisolism, being particularly useful in MACS-positive tumors that otherwise do not present the typical clinical picture of Cushing’s syndrome, but potentially associate various cardio-metabolic complications, as mentioned [96,97]. Moreover, serum DHEAS levels diminish with aging and are higher in males vs. females. As found in the present analysis, patients with MACS have significantly lower levels of DHEAS than subjects without MACS, owing to the relative suppression of ACTH [98], which itself might help MACS diagnosis in cases with low or low-normal baseline values [99].

Of note, the role of imaging tools should be remarked, as well, in MACS characterization among AIs [100]. Recent studies have suggested that radiomic analysis of native computed tomography captures, using texture parameters, could effectively identify patients with MACS among those with AIs, offering an elegant non-invasive screening tool [101,102].

### 4.2. From DST to MACS: A Modern Pathway, a Traditional Road

The importance of correct AIs description/characterization with respect to the hormonal profile includes: a prompt identification of MACS-positive subgroup, understanding the relationship with cardio-metabolic complications (amid transversal and longitudinal DST analysis), an adequate selection of surgery candidates, and the decision of re-assessment in terms of timing and protocol of assays in individuals who are not referred to adrenalectomy. DST represents the most useful tool in assisting the hormonal description since post-DST cortisol values are connected to all these mentioned aspects [34,35,36,37,38,39,50,51,52,53,54,77,78]. Moreover, even non-MACS subgroups (those with a cortisol level below 1.8 µg/dL) associate a certain level of disease burden. For instance, while Güneş et al. [39] found that a level ≥ 0.87 µg/dL was statistically significant associated with a higher prevalence of hypertension in patients with AIs (OR = 3.24, *p* = 0.047) [39], Favero et al. [37] identified a level between 1.2 and 1.79 µg/dL that was more likely to associate hypertension (OR = 1.55), diabetes (OR = 1.60), and both conditions (OR = 1.96), after adjustment for confounders [37]. Of note, the risk of diabetes seems even higher in patients co-confirmed with MACS and primary aldosteronism (Connshing syndrome) via a dual hormonal interference [55,103,104,105], but this was out of our scope.

**Figure 3:**
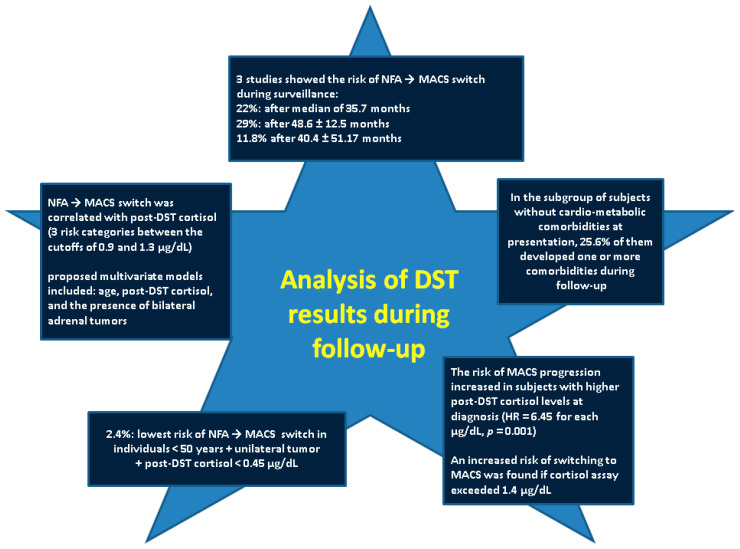
Longitudinal analysis: main findings (Abbreviations: DST = dexamethasone suppression test, HR = hazard ratio, MACS = mild autonomous cortisol secretion, NFA = non-functional adrenal adenoma).

Recently, patients with MACS-positive adrenal tumors were found to exhibit increased choroidal thickness and a higher frequency of pachychoroid pigment epitheliopathy compared to healthy individuals, suggesting that choroidal thickening could serve as a novel biomarker for assessing the panel of related comorbidities [106]. Additionally, serum Fibroblast Growth Factor (FGF) 21 levels were found to positively correlate with post-DST cortisol, cortisol-to-ACTH ratio, and tumor size, suggesting a potential role for FGF21 as a biomarker of the cortisol secretion severity [107]. An alternative and promising predictor for differentiating MACS from NFAs is represented by the Classification and Regression Trees Model, which, by analyzing a combination of leukocyte-related parameters, demonstrates a significant improvement in diagnosis accuracy, offering a robust tool to assist practitioners in the clinical decision-making [108]. Furthermore, the mentioned data regarding certain subgroups of patients with NFAs (cortisol below 1.8 µg/dL) that might present some level of hypercortisolism and benefit from adrenalectomy in terms of blood pressure and glucose metabolism improvement represents an argument for re-considering long-term interventional strategies [109,110,111]. Of note, developing post-operative hypocortisolism, while initially MACS profile was not confirmed, also, contributes to re-thinking the picture of the hormonal imbalance in non-MACS subgroups [46,112] (Figure 3).

### 4.3. Current Limits and Further Research

The limitations of the current work are represented by the narrative design. We intended to analyze non-restricted (non-systematic) variables/parameters as they are provided by the current studies which greatly vary in terms of design, resources, and protocol. We considered that a broader perspective might cover the mentioned DST spectrum outside the guideline-based approach. The sample size was limited by the single database and timing of search, which was meant to offer an update to the most recent findings in an otherwise traditional topic. Novel approaches such as applying new post-DST cortisol cutoffs, multimodal models of prediction and positioning additional novel biomarkers in daily practice are currently under development and we expect a multilayered expansion of this area due to the increasing number of AIs detection and prolonged lifespan. Notably, the (potential) cortisol excess in AIs might also associate an elevated osteoporotic fracture risk and other osseous complications [113,114,115,116], which were out of our scope. They represent, as well, another important contributor to the spectrum of AIs-related complications (which also includes a higher risk of kidney stones, renal function impairment, cognitive dysfunction, infectious disorders, depression, memory loss, reproductive issues, and even fatty liver disease in some cases, etc. [117,118,119,120,121,122,123]. Finally, as already specified, concomitant primary aldosteronism in MACS-positive tumors represents a novel clinical entity. This particular form of hormonal excess is unceasingly recognized, although the criteria for defining cortisol excess vary among studies, as does the reported prevalence. For instance, in a recent study, using a post-DST cortisol cutoff of >1.8 µg/dL, mild hypercortisolism was identified in nearly 30% of patients with primary aldosteronism [103,104,105,124,125]. Which is the true effect of this dual hormonal hit, and the adequate testing for MACS under these circumstances, and also the direct/indirect contribution to the cardio-metabolic outcomes remains an open issue.

## 5. Conclusions

The importance of exploring the domain of AIs/NFAs/MACS relates to an increasing detection in aging population, hence, the importance of their optimum hormonal characterization and identifying/forestalling cardio-metabolic consequences. DST remains the key tool for identifying MACS amid AIs. Post-DST cortisol serves as an independent predictor of cardio-metabolic outcomes, kidney dysfunction, while adrenalectomy may correct them in both MACS and NFAs, especially in younger population. Moreover, it serves as a predictor of switching the NFA into MACS category during surveillance. Additional biochemical tests, such as ACTH, and DHEAS (and to a lesser extend 24-h UFC, and LNSC) provide valuable supplementary insights, though they are not standardized. These tests can enhance diagnosis accuracy, help the evaluation of the cardio-metabolic risk, and refine the follow-up strategy. Given the variability in their use and the potential for progression of the hormonal category, further research is needed to better define the role of these diagnosis tools and improve patients’ management. Changing the hormonal behavior over time implies awareness, since it increases the overall disease burden.

## Figures and Tables

**Table 1:** Included studies that provided and analysis of post-DST cortisol cutoffs and their association with cardio-metabolic and renal outcomes [34,35,36,37,38,39]; (the studies display starts with the most recent publication date).

First Author Year of Publication Reference Number	Study Design	Number of Patients Sex Ratio (F/M) Age (Years)	Outcomes
Bonaventura 2024 [34]	Retrospective	N = 32 AI F/M = 19/13 Age median (IQR) = 61 (51–66) y N = 25 with MACS (1 mg DST > 1.8 µg/dL) F/M = 14/11 Age median (IQR) = 58 (9–66) y N = 7 with NFA (1 mg DST ≤ 1.8 µg/dL) F/M = 5/2 Age median (IQR) = 66 (64–67)	Preoperative cortisol after 1 mg-DST (4.75 µg/dL) as the only significant predictor of 6-week adrenal recovery following adrenalectomy Sensitivity = 89.5% Specificity = 72.7% AUC = 0.87, *p* < 0.001 Diabetes was associated with a significantly reduced chance of post-surgery recovery (OR = 24.55, *p* = 0.036).
Olsen 2023 [35]	Cross-sectional	N = 631 with AI (1 mg DST <1.8 µg/dL) F/M = 352/279 Age median (IQR) = 63.2 (55.1–69.7) y N = 516 with AI (1 mg DST ≥ 1.8 µg/dL) F/M = 322/194 Age median (IQR) = 67.3 (61.0–74.4) y	Negative correlation between 1 mg DST and eGFR, with 1 mg DST increasing by 9% (95% CI: 6–11%) for each 10 mL/min/1.73 m^2^ decrease in eGFR
Rahimi 2023 [36]	Cohort	N = 972 with AI F/M = 629/343 Age median (IQR) = 60.9 (52.6–68.7) y N = 429 with MACS (1 mg DST ≥ 1.8 µg/dL) F/M = 285/144 Age median (IQR)= 62.8 (54.6–71.1) y N = 543 with NFA (1 mg DST < 1.8 µg/dL) F/M = 344/199 Age median (IQR)= 59.4 (50.5–67.3) y	eGFR MACS vs. NFA: 79.6 vs. 83.8 mL/min/1.73 m^2^, *p* < 0.001) Higher 1 mg DST cortisol levels were independently associated with a decline in kidney function, each doubling of cortisol was linked to a decrease of 1.01 mL/min/1.73 m^2^ in eGFR (*p* = 0.017), even after adjusting for age (−7.94; *p* = 0.001) and hypertension (−2.72; *p* = 0.038), highlighting the role of subtle cortisol excess in renal impairment.
Favero 2023 [37]	Retrospective cross-sectional	N = 615 F/M = 289/326 Age (mean ± SD) = 60.15 ± 11.8 y N = 289 NFA (1 mg DST < 1.2 µg/dL) F/M = 182/107 Age (mean ± SD) = 57.5 ± 12.3 y N = 326 NFA (1 mg DST ≥ 1.2 µg/dL) F/M = 195/131 Age (mean ± SD) = 62.5 ± 10.9 y	1 mg DST 1.2 µg/dL was the cutoff with the highest accuracy in identifying patients with either hypertension or diabetes mellitus: AUC = 0.604 (95% CI: 0.560–0.649) Sensitivity = 60.2% Specificity = 56.0% Hypertension or diabetes mellitus AUC = 0.611 (95% CI: 0.545–0.675) Sensitivity = 60.4% Specificity = 69.6% Compared to patients with 1 mg DST below 1.2 µg/dL (N = 289) vs. 1.2–1.79 µg/dL (N = 326) had: Lower ACTH levels (15.3 ± 10.1 vs. 17.7 ± 11.9 pg/mL, *p* = 0.008), Older age (62.5 ± 10.9 vs. 57.5 ± 12.3 y, *p* < 0.001) Higher prevalence of: Hypertension (52.5% vs. 38.1%, *p* < 0.001) Diabetes mellitus (23.3% vs. 13.1%, *p* = 0.001) Both hypertension and diabetes mellitus (16.9% vs. 8.3%, *p* < 0.002) Cardiovascular events (7.3% vs. 3.2%, *p* = 0.028) After adjusting for confounders (age, gender, obesity, dyslipidemia, and either hypertension or diabetes mellitus), 1 mg DST levels between 1.2–1.79 µg/dL remained significantly associated with: Hypertension (OR = 1.55, 95% CI: 1.08–2.23, *p* = 0.018) Diabetes mellitus (OR = 1.60, 95% CI: 1.01–2.57, *p* = 0.045) Both hypertension and diabetes mellitus (OR = 1.96, 95% CI: 1.12–3.41, *p* = 0.018)
Araujo-Castro 2023 [38]	Retrospective	N = 593 NFA F/M = 343/250 Age (mean ± SD) = 62.3 ± 10.83 y N = 442 NFA (1 mg DST ≤ 1.4 µg/dL) F/M = 257/185 Age (mean ± SD) = 61.3 ± 10.42 y N = 151 NFA (1 mg-DST > 1.4 µg/dL) F/M = 86/65 Age (mean ± SD) = 64.9 ± 11.58 y N = 412 NFA (1 mg DST ≤ 0.9 µg/dL) F/M = 241/171 Age (mean ± SD) = 59.6 ± 10.79 y N = 181 NFA (1 mg-DST > 0.9 µg/dL) F/M = 104/77 Age (mean ± SD) = 63.4 ± 10.66 y	1 mg DST 0.9 µg/dL threshold proves to be useful in identifying patients with NFA at higher cardiovascular risk OR = 2.23 (1.10–4.53)
Güneş 2023 [39]	Retrospective	N = 123 with AI F/M = 90/33 Age (mean ± SD) = 53.0 ± 10.9 N = 114 controls F/M = 91/23 Age (mean ± SD) = 52.9 ± 7.4 y	ROC analysis identified the optimal 1 mg DST level for HT, which was 0.87 μg/dL. HT by DST level: <0.87 μg/dL → 42.6% ≥0.87 μg/dL → 66.1%, *p* = 0.009 Independent predictors of HT (binary logistic regression): Age: β = 0.068, OR = 1.07 (95% CI: 1.02–1.12), *p* = 0.004 DST level: β = 1.18, OR = 3.24, 95% CI: 1.02–10.34, *p* = 0.047

Abbreviation: ACS = autonomous cortisol secretion, ACTH = adrenocorticotropic hormone, AI = adrenal incidentalomas, AUC = area under the curve, CI confidence interval, eGFR = estimated glomerular filtration rate, F = female, HPA = hypothalamic-pituitary-adrenal axis, HR = hazard ratio, IQR = interquartile range, M = male, MACS = mild autonomous cortisol secretion, N = number of patients, NFA = non-functioning adrenal adenoma, OR = odds ratio, ROC= Receiver Operating Characteristic, SD = standard deviation, vs. = versus, y = years (red font = the blood cortisol cutoff following dexamethasone administration during dexamethasone suppression test).

**Table 2:** Included studies that evaluated the potential improvements of DST through the addition of other hormonal markers (ACTH, UFC, LNSC, DHEAS) in order to pinpoint MACS [50,51,52,53,54]; (the display starts with the most recent publication date).

First Author Year of Publication Reference Number	Study Design	Number of Patients Sex Ratio (F/M) Age (Years)	Outcomes
Turan Erdogan 2024 [50]	Retrospective study	N = 461 with AI F/M = 309/152 Age (mean ± SD) = 54.8 ± 10.19 y N = 77 with MACS (1 mg DST > 1.8 µg/dL) F/M = 56/21 Age (mean ± SD) = 56.87 ± 10.67 y N = 384 with NFA (1 mg DST ≤ 1.8 µg/dL) F/M = 253/131 Age (mean ± SD) = 54.39 ± 10.05 y	Predicted MACS: DHEAS ≤ 49.31 µg/dL Sensitivity = 61% Specificity = 73% AUC = 0.704 (95% CI: 0.636–0.771, *p* < 0.001)
Efthymiadis 2024 [51]	Retrospective study	N = 53 with CS of these 24 with MACS (1 mg DST > 1.8 µg/dL) and 27 with Cushing disease F/M = 42/11 Age (mean ± SD) = 56 ± 16 y	MACS 1 mg DST (cutoff 1.8 µg/dL) Sensitivity = 100% (95% CI: 82.4–100.0) Specificity = 52.2% (95% CI: 30.6–73.2) AUC = 0.76 (95% CI: 0.66–1.00, *p* = 0.004) LNSC (cutoff 1.7 nmol/L) Sensitivity = 77.2% (95% CI: 54.6–92.2) Specificity = 64.8% (95% CI: 47.5–79.8) AUC = 0.71 (95% CI: 0.574–0.848, *p* = 0.007) LDDST (cutoff 1.8 µg/dL) Sensitivity = 93.8% (95% CI: 69.8–99.8) Specificity = 72.7% (95% CI: 39.0–94.0) AUC = 0.83 (95% CI: 0.66–1.00, *p* = 0.004) UFC (cutoff 135 nmol/L) Sensitivity = 17.7% (95% CI: 3.8–43.4) Specificity = 58.8% (95% CI: 32.9–81.6) AUC = 0.62 (95% CI: 0.43–0.80, *p* = 0.242) LNSE (cutoff 15.2 nmol/L) Sensitivity = 27.8% (95% CI: 9.7–53.5) Specificity = 96.1% (95% CI: 80.4–99.9) AUC = 0.66 (95% CI: 0.47–0.85, *p* = 0.102) Combining these with ACTH > 12.6 pmol/L as cutoff distinguishing Cushing disease from MACS Sensitivity = 100% Specificity = 86.7% AUC = 0.98 (95% CI: 0.87–1.00, *p* < 0.001)
Al-Waeli 2023 [52]	Cross-sectional study	N = 38 with AI of these 5 were diagnosed with MACS (1 mg DST > 1.8 µg/dL) F/M = 23/15 Age (mean ± SD) = 47.6 ± 18.3 y	DHEAS ≤ 75 µg/dL Sensitivity = 80% Specificity = 73.3% Negative predictive values = 95.7% Positive predictive values = 33.3% DHEAS ratio ≤ 1.7 Sensitivity = 80% Specificity = 76.6% Negative predictive values = 95.8% Positive predictive values = 36.4%
Araujo-Castro, 2023 [53]	Cross-sectional study	N = 49 AI ACS = 25 (1 mg DST > 1.8 µg/dL) F/M = 17/8 Age (mean ± SD) = 67.4 ± 9.68 y NFA = 24 (1 mg DST ≤ 1.8 µg/dL) F/M = 16/8 Age (mean ± SD) = 70.2 ± 7.83 y	ACS-related comorbidities were moderately accurately predicted by post-DST cortisol alone: AUC = 0.767 (95% CI: 0.634–0.882) Post-DST cortisol + urinary cortisone, α-cortol, and THS provided the highest diagnosis accuracy: AUC = 0.813 (95% CI: 0.680–0.912) Post-DST cortisol + glucocorticoid metabolites + DHEAS: AUC = 0.853 (95% CI: 0.712–0.954)
Issa 2023 [54]	Retrospective study	N = 173 with AI F/M = 96/77 Age (mean ± SD) = 64.2 ± 11.3 y	Correlation between 1 mg DST salivary cortisone and serum cortisol with an *r* = 0.95 (*p* < 0.001) Sensitivity = 83.3% Specificity = 91.4% Accuracy = 88.2% Four predictive parameters: post-dexamethasone salivary cortisone, baseline serum cortisol, the salivary cortisone suppression ratio (pre-/post-dexamethasone), and sex yielded a: Sensitivity = 88.5% Specificity = 91.2% Salivary cortisone alone (cutoff < 2.7 nmol/L) for predicting a 1 mg DST ≤ 1.8 µg/dL: Sensitivity = 85.3% Specificity = 91.7%

Abbreviation: ACS = autonomous cortisol secretion, ACTH = adrenocorticotropic hormone, AI = adrenal incidentalomas, AUC = area under the curve, CI = confidence interval, DHEAS = dehydroepiandrosterone sulfate, eGFR = estimated glomerular filtration rate, F = female, HPA = hypothalamic-pituitary-adrenal axis, HR = hazard ratio, IQR = interquartile range, LDDST = low-dose dexamethasone suppression test, LNSC = late-night salivary cortisol, LNSE = late-night salivary cortisone, M = male, MACS = mild autonomous cortisol secretion, N = number of patients, NFA = non-functioning adrenal adenoma, OR = odds ratio, ROC= Receiver Operating Characteristic, SD = standard deviation, vs. = versus, Y = years.

**Table 3:** Studies assessing longitudinal variations in DST results and changing of the NFA/MACS category [38,77,78].

First Author Year of Publication Reference Number	Study Design	Number of Patients Sex Ratio (F/M) Age (Years)	Outcomes
Petramala 2024 [77]	Retrospective study	N = 132 with AI F/M = 76/56 Age (mean ± SD) = 61.7 ± 10.8 y N = 90 AI (1 mg DST < 1.8 µg/dL) F/M = 27/17 Age (mean ± SD) = 61.6 ± 11.5 y N = 43 AI (1 mg DST > 1.8 µg/dL) F/M = 11/11 Age (mean ± SD) = 61.8 ± 9.4 y	Follow-up (annually for at least 5 years): 29.2% of subjects developed MACS (1 mg DST > 1.8 µg/dL) At the end of follow-up, MACS patients showed higher diastolic blood pressure values: NFA vs. MACS: 81.6 ± 10.5 vs. 83.7 ± 9.7 mmHg, *p* < 0.05
Araujo-Castro 2023 [78]	Retrospective study	N = 331 with NFA F/M = 197/134 Age (mean ± SD) = 62.0 ± 10.6 y During a median follow-up time of 35.7 months N = 73 (22.1%) develop ACS (1 mg DST > 1.8 µg/dL)	The greatest predictor of ACS development during follow-up was a combination of age, post-DST serum cortisol, and bilaterality at presentation, which demonstrated good diagnosis accuracy AUC = 0.70 (95% CI: 0.64–0.76) DST being the threshold of 1.3 µg/dL for the prediction of ACS development AUC = 0.701 (95% CI: 0.637–0.765) Sensitivity = 70% Specificity = 62% Positive predictive value = 0.37% Negative predictive value = 99% The lowest probability of developing ACS: patients under 50 with cortisol post-DST values < 0.45 µg/dL and unilateral tumors had (2.42%) Baseline post-DST serum cortisol levels at diagnosis were significantly associated with the development of ACS throughout follow-up (hazard ratio 3.56 for each µg/dL, *p* < 0.001) Follow-up 1 mg DST < 0.9 µg/dL, follow-up is probably unnecessary 1 mg DST 0.9–1.3 µg/dL, repeating the DST every 2–3 years for five 1 mg DST > 1.3 µg/dL requires annual re-evaluation for at least five years
Araujo-Castro 2023 [38]	Retrospective study	N = 593 NFA F/M = 343/250 Age (mean ± SD) = 62.3 ± 10.83 y N = 442 NFA (1 mg DST ≤ 1.4 µg/dL) F/M = 257/185 Age (mean ± SD) = 61.3 ± 10.42 y N = 151 NFA (1 mg-DST > 1.4 µg/dL) F/M = 86/65 Age (mean ± SD) = 64.9 ± 11.58 y N = 412 NFA (1 mg DST ≤ 0.9 µg/dL) F/M = 241/171 Age (mean ± SD) = 59.6 ± 10.79 y N = 181 NFA (1 mg-DST > 0.9 µg/dL) F/M = 104/77 Age (mean ± SD) = 63.4 ± 10.66 y	Follow-up of 40.4 ± 51.17 months, 11.8% of the patients developed ACS ACS was increased in patients with higher blood cortisol post-DST levels (HR = 6.45 for each µg/dL, *p* = 0.001) Increased risk of ACS development when the DST level exceeded 1.4 µg/dL

Abbreviation: ACS = autonomous cortisol secretion, AI = adrenal incidentalomas, AUC = area under the curve, CI = confidence interval, F = female, HPA = hypothalamic-pituitary-adrenal axis, HR = hazard ratio, IQR = interquartile range, M = male, MACS = mild autonomous cortisol secretion, N = number of patients, NFA = non-functioning adrenal adenoma, OR = odds ratio, SD = standard deviation, vs. = versus, y = years.

## Data Availability

No new data were created.

## Abbreviations

The following abbreviations are used in this manuscript:

ACTH	adrenocorticotropic hormone
ACS	autonomous cortisol secretion
AI	adrenal incidentaloma
AUC	area under the curve
BMI	body mass index
CI	confidence interval
DHEAS	dehydroepiandrosterone sulfate
DST	1-mg dexamethasone suppression test
eGFR	estimated glomerular filtration rate
F	female
FGF	Fibroblast Growth Factor
HPA	hypothalamic-pituitaryadrenal
HR	hazard ratio
IQR	interquartile range
LNSC	late-night salivary cortisol
LNSE	late-night salivary cortisone
LDDST	low-dose dexamethasone suppression test
MACS	mild autonomous cortisol secretion
M	male
NFA	non-functioning adrenal adenomas
n	number of studies
N	number of patients
OR	odds ratio
ROC	Receiver Operating Characteristic
SD	standard deviation
THF	tetrahydrocortisol
THS	tetrahydro-11-deoxycortisol
THE	tetrahydrocortisone
UFC	urinary free cortisol
vs.	versus
y	years
